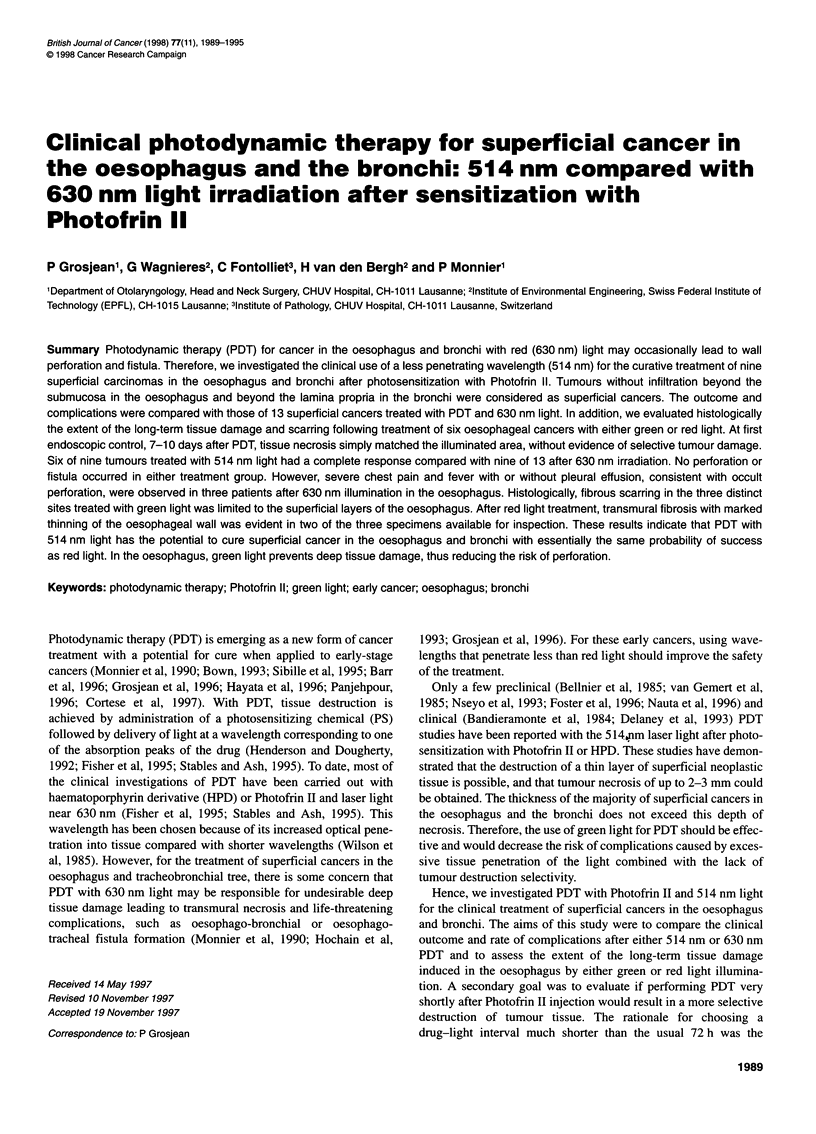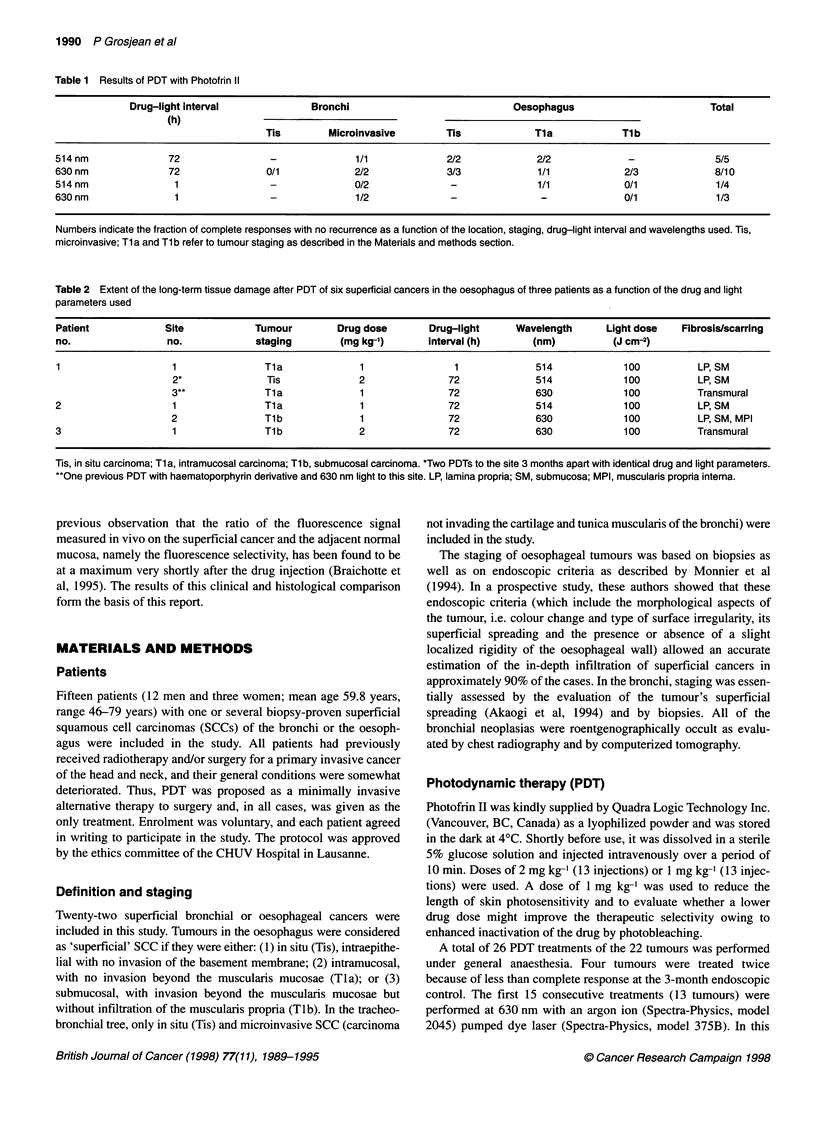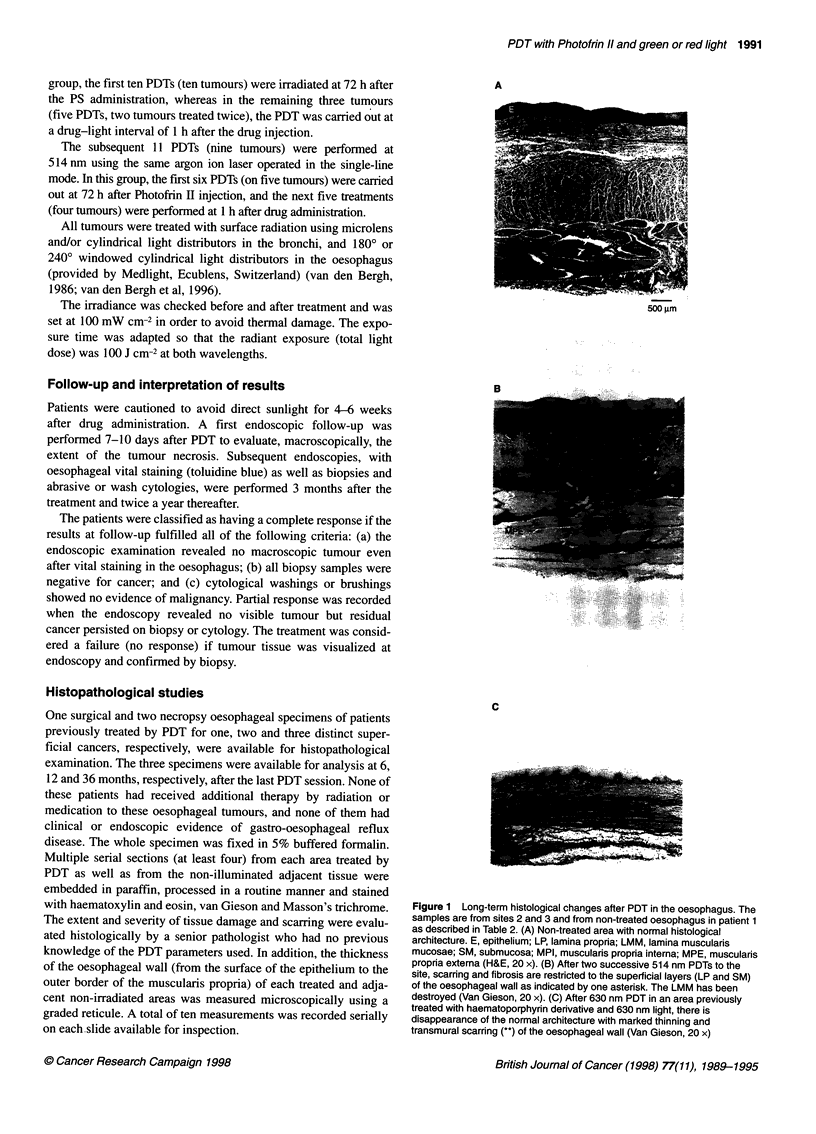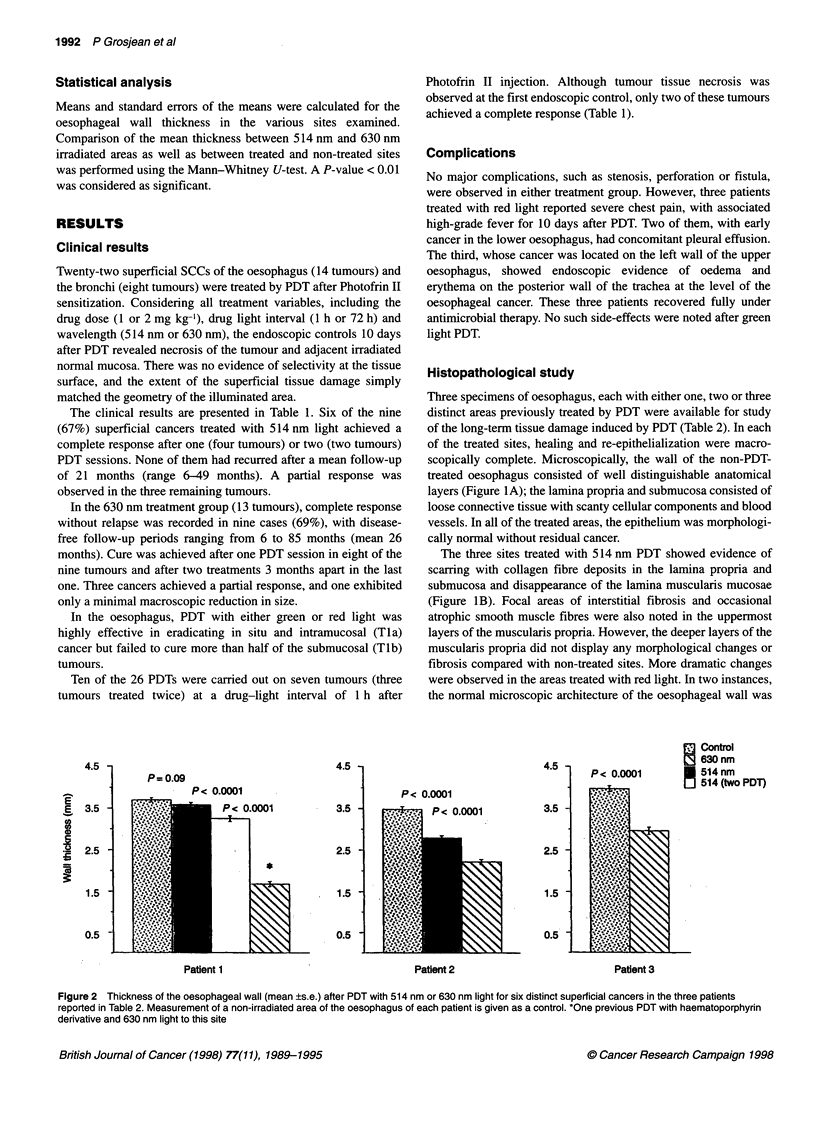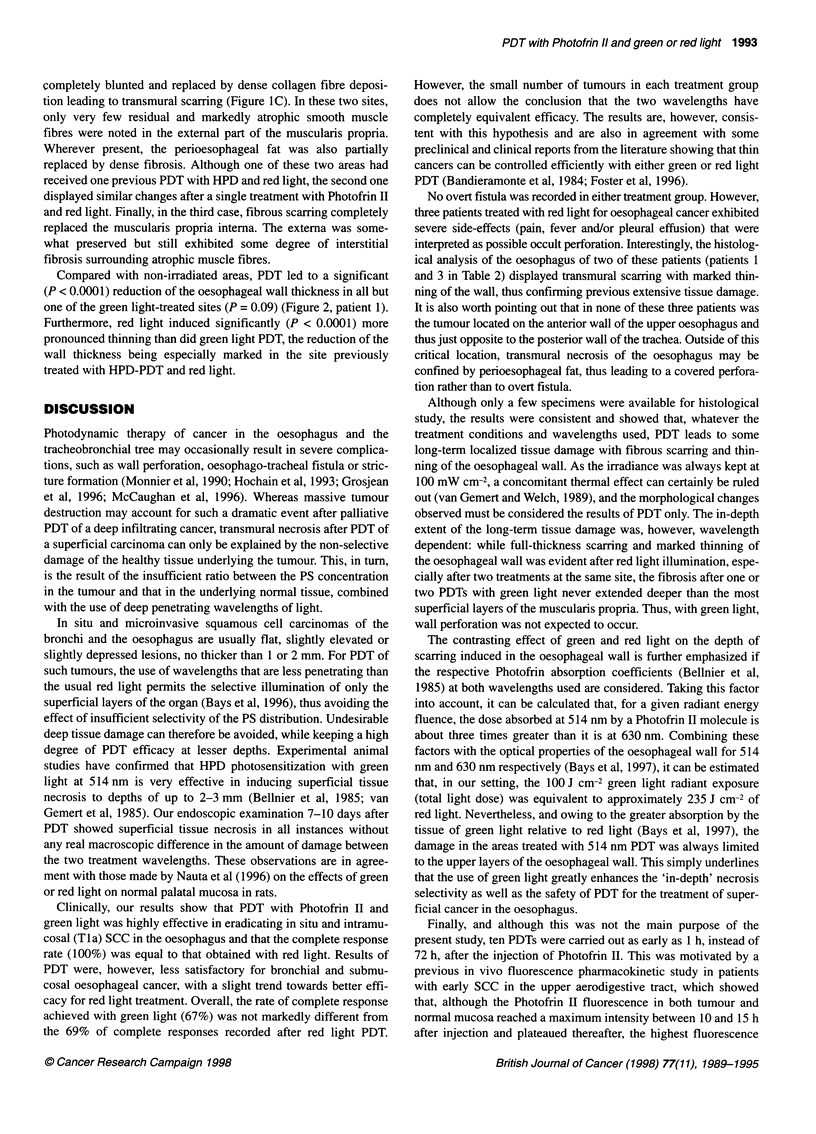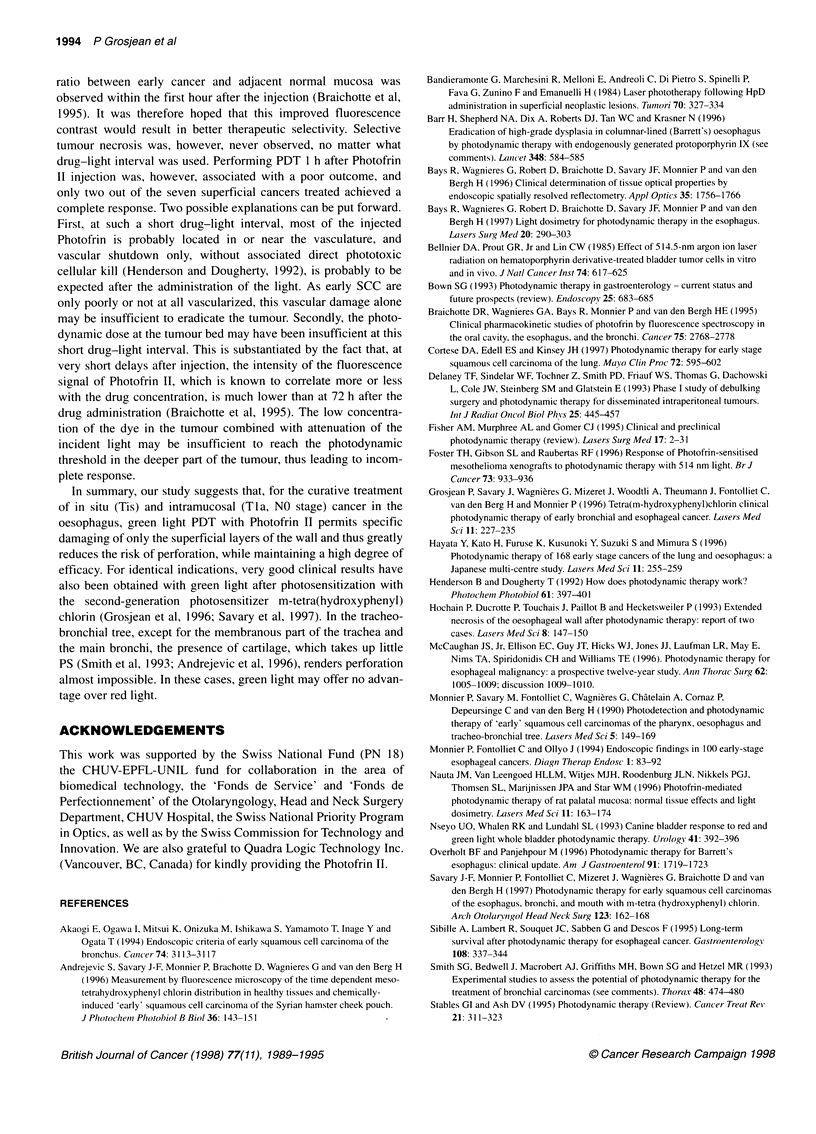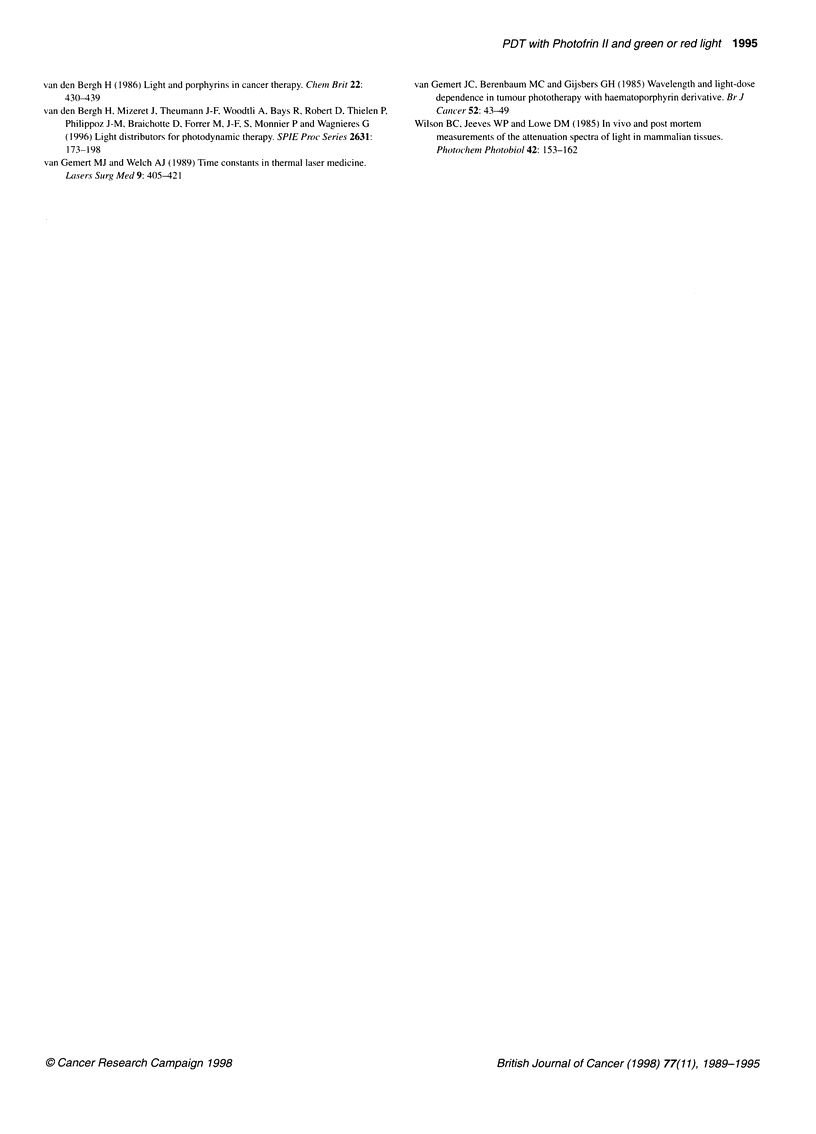# Clinical photodynamic therapy for superficial cancer in the oesophagus and the bronchi: 514 nm compared with 630 nm light irradiation after sensitization with Photofrin II.

**DOI:** 10.1038/bjc.1998.330

**Published:** 1998-06

**Authors:** P. Grosjean, G. Wagnieres, C. Fontolliet, H. van den Bergh, P. Monnier

**Affiliations:** Department of Otolaryngology, Head and Neck Surgery, CHUV Hospital, Lausanne, Switzerland.

## Abstract

**Images:**


British Journal of Cancer (1998) 77(11), 1989–1995
© 1998 Cancer Research Campaign

### Clinical photodynamic therapy for superficial cancer in the oesophagus and the bronchi: 514 nm compared with 630 nm light irradiation after sensitization with Photofrin II

**P Grosjean[1], G Wagnieres[2], C Fontolliet[3], H van den Bergh[2] and P Monnier[1]**

[1]Department of Otolaryngology, Head and Neck Surgery, CHUV Hospital, CH-1011 Lausanne; [2]Institute of Environmental Engineering, Swiss Federal Institute of Technology (EPFL), CH-1015 Lausanne; [3]Institute of Pathology, CHUV Hospital, CH-1011 Lausanne, Switzerland

**Summary** Photodynamic therapy (PDT) for cancer in the oesophagus and bronchi with red (630 nm) light may occasionally lead to wall perforation and fistula. Therefore, we investigated the clinical use of a less penetrating wavelength (514 nm) for the curative treatment of nine superficial carcinomas in the oesophagus and bronchi after photosensitization with Photofrin II. Tumours without infiltration beyond the submucosa in the oesophagus and beyond the lamina propria in the bronchi were considered as superficial cancers. The outcome and complications were compared with those of 13 superficial cancers treated with PDT and 630 nm light. In addition, we evaluated histologically the extent of the long-term tissue damage and scarring following treatment of six oesophageal cancers with either green or red light. At first endoscopic control, 7–10 days after PDT, tissue necrosis simply matched the illuminated area, without evidence of selective tumour damage. Six of nine tumours treated with 514 nm light had a complete response compared with nine of 13 after 630 nm irradiation. No perforation or fistula occurred in either treatment group. However, severe chest pain and fever with or without pleural effusion, consistent with occult perforation, were observed in three patients after 630 nm illumination in the oesophagus. Histologically, fibrous scarring in the three distinct sites treated with green light was limited to the superficial layers of the oesophagus. After red light treatment, transmural fibrosis with marked thinning of the oesophageal wall was evident in two of the three specimens available for inspection. These results indicate that PDT with 514 nm light has the potential to cure superficial cancer in the oesophagus and bronchi with essentially the same probability of success as red light. In the oesophagus, green light prevents deep tissue damage, thus reducing the risk of perforation.

**Keywords:** photodynamic therapy; Photofrin II; green light; early cancer; oesophagus; bronchi

Photodynamic therapy (PDT) is emerging as a new form of cancer treatment with a potential for cure when applied to early-stage cancers (Monnier et al, 1990; Bown, 1993; Sibille et al, 1995; Barr et al, 1996; Grosjean et al, 1996; Hayata et al, 1996; Panjehpour, 1996; Cortese et al, 1997). With PDT, tissue destruction is achieved by administration of a photosensitizing chemical (PS) followed by delivery of light at a wavelength corresponding to one of the absorption peaks of the drug (Henderson and Dougherty, 1992; Fisher et al, 1995; Stables and Ash, 1995). To date, most of the clinical investigations of PDT have been carried out with haematoporphyrin derivative (HPD) or Photofrin II and laser light near 630 nm (Fisher et al, 1995; Stables and Ash, 1995). This wavelength has been chosen because of its increased optical penetration into tissue compared with shorter wavelengths (Wilson et al, 1985). However, for the treatment of superficial cancers in the oesophagus and tracheobronchial tree, there is some concern that PDT with 630 nm light may be responsible for undesirable deep tissue damage leading to transmural necrosis and life-threatening complications, such as oesophago-bronchial or oesophago-tracheal fistula formation (Monnier et al, 1990; Hochain et al, 1993; Grosjean et al, 1996). For these early cancers, using wavelengths that penetrate less than red light should improve the safety of the treatment.

*Received 14 May 1997*
*Revised 10 November 1997*
*Accepted 19 November 1997*

*Correspondence to:* P Grosjean

Only a few preclinical (Bellnier et al, 1985; van Gemert et al, 1985; Nseyo et al, 1993; Foster et al, 1996; Nauta et al, 1996) and clinical (Bandieramonte et al, 1984; Delaney et al, 1993) PDT studies have been reported with the 514 nm laser light after photosensitization with Photofrin II or HPD. These studies have demonstrated that the destruction of a thin layer of superficial neoplastic tissue is possible, and that tumour necrosis of up to 2–3 mm could be obtained. The thickness of the majority of superficial cancers in the oesophagus and the bronchi does not exceed this depth of necrosis. Therefore, the use of green light for PDT should be effective and would decrease the risk of complications caused by excessive tissue penetration of the light combined with the lack of tumour destruction selectivity.

Hence, we investigated PDT with Photofrin II and 514 nm light for the clinical treatment of superficial cancers in the oesophagus and bronchi. The aims of this study were to compare the clinical outcome and rate of complications after either 514 nm or 630 nm PDT and to assess the extent of the long-term tissue damage induced in the oesophagus by either green or red light illumination. A secondary goal was to evaluate if performing PDT very shortly after Photofrin II injection would result in a more selective destruction of tumour tissue. The rationale for choosing a drug–light interval much shorter than the usual 72 h was the

Table 1 Results of PDT with Photofrin II

| | Drug–light interval (h) | Bronchi | | Oesophagus | | | Total |
|---|---|---|---|---|---|---|---|
| | | Tis | Microinvasive | Tis | T1a | T1b | |
| 514 nm | 72 | – | 1/1 | 2/2 | 2/2 | – | 5/5 |
| 630 nm | 72 | 0/1 | 2/2 | 3/3 | 1/1 | 2/3 | 8/10 |
| 514 nm | 1 | – | 0/2 | – | 1/1 | 0/1 | 1/4 |
| 630 nm | 1 | – | 1/2 | – | – | 0/1 | 1/3 |

Numbers indicate the fraction of complete responses with no recurrence as a function of the location, staging, drug–light interval and wavelengths used. Tis, microinvasive; T1a and T1b refer to tumour staging as described in the Materials and methods section.

Table 2 Extent of the long-term tissue damage after PDT of six superficial cancers in the oesophagus of three patients as a function of the drug and light parameters used

| Patient no. | Site no. | Tumour staging | Drug dose (mg $kg^{-1}$) | Drug–light interval (h) | Wavelength (nm) | Light dose (J $cm^{-2}$) | Fibrosis/scarring |
|---|---|---|---|---|---|---|---|
| 1 | 1 | T1a | 1 | 1 | 514 | 100 | LP, SM |
| | 2* | Tis | 2 | 72 | 514 | 100 | LP, SM |
| | 3** | T1a | 1 | 72 | 630 | 100 | Transmural |
| 2 | 1 | T1a | 1 | 72 | 514 | 100 | LP, SM |
| | 2 | T1b | 1 | 72 | 630 | 100 | LP, SM, MPI |
| 3 | 1 | T1b | 2 | 72 | 630 | 100 | Transmural |

Tis, in situ carcinoma; T1a, intramucosal carcinoma; T1b, submucosal carcinoma. *Two PDTs to the site 3 months apart with identical drug and light parameters. **One previous PDT with haematoporphyrin derivative and 630 nm light to this site. LP, lamina propria; SM, submucosa; MPI, muscularis propria interna.

previous observation that the ratio of the fluorescence signal measured in vivo on the superficial cancer and the adjacent normal mucosa, namely the fluorescence selectivity, has been found to be at a maximum very shortly after the drug injection (Braichotte et al, 1995). The results of this clinical and histological comparison form the basis of this report.

### MATERIALS AND METHODS

### Patients

Fifteen patients (12 men and three women; mean age 59.8 years, range 46–79 years) with one or several biopsy-proven superficial squamous cell carcinomas (SCCs) of the bronchi or the oesophagus were included in the study. All patients had previously received radiotherapy and/or surgery for a primary invasive cancer of the head and neck, and their general conditions were somewhat deteriorated. Thus, PDT was proposed as a minimally invasive alternative therapy to surgery and, in all cases, was given as the only treatment. Enrolment was voluntary, and each patient agreed in writing to participate in the study. The protocol was approved by the ethics committee of the CHUV Hospital in Lausanne.

### Definition and staging

Twenty-two superficial bronchial or oesophageal cancers were included in this study. Tumours in the oesophagus were considered as 'superficial' SCC if they were either: (1) in situ (Tis), intraepithelial with no invasion of the basement membrane; (2) intramucosal, with no invasion beyond the muscularis mucosae (T1a); or (3) submucosal, with invasion beyond the muscularis mucosae but without infiltration of the muscularis propria (T1b). In the tracheobronchial tree, only in situ (Tis) and microinvasive SCC (carcinoma not invading the cartilage and tunica muscularis of the bronchi) were included in the study.

The staging of oesophageal tumours was based on biopsies as well as on endoscopic criteria as described by Monnier et al (1994). In a prospective study, these authors showed that these endoscopic criteria (which include the morphological aspects of the tumour, i.e. colour change and type of surface irregularity, its superficial spreading and the presence or absence of a slight localized rigidity of the oesophageal wall) allowed an accurate estimation of the in-depth infiltration of superficial cancers in approximately 90% of the cases. In the bronchi, staging was essentially assessed by the evaluation of the tumour's superficial spreading (Akaogi et al, 1994) and by biopsies. All of the bronchial neoplasias were roentgenographically occult as evaluated by chest radiography and by computerized tomography.

### Photodynamic therapy (PDT)

Photofrin II was kindly supplied by Quadra Logic Technology Inc. (Vancouver, BC, Canada) as a lyophilized powder and was stored in the dark at 4°C. Shortly before use, it was dissolved in a sterile 5% glucose solution and injected intravenously over a period of 10 min. Doses of 2 mg $kg^{-1}$ (13 injections) or 1 mg $kg^{-1}$ (13 injections) were used. A dose of 1 mg $kg^{-1}$ was used to reduce the length of skin photosensitivity and to evaluate whether a lower drug dose might improve the therapeutic selectivity owing to enhanced inactivation of the drug by photobleaching.

A total of 26 PDT treatments of the 22 tumours was performed under general anaesthesia. Four tumours were treated twice because of less than complete response at the 3-month endoscopic control. The first 15 consecutive treatments (13 tumours) were performed at 630 nm with an argon ion (Spectra-Physics, model 2045) pumped dye laser (Spectra-Physics, model 375B). In this

 © Cancer Research Campaign 1998

group, the first ten PDTs (ten tumours) were irradiated at 72 h after the PS administration, whereas in the remaining three tumours (five PDTs, two tumours treated twice), the PDT was carried out at a drug–light interval of 1 h after the drug injection.

The subsequent 11 PDTs (nine tumours) were performed at 514 nm using the same argon ion laser operated in the single-line mode. In this group, the first six PDTs (on five tumours) were carried out at 72 h after Photofrin II injection, and the next five treatments (four tumours) were performed at 1 h after drug administration.

All tumours were treated with surface radiation using microlens and/or cylindrical light distributors in the bronchi, and 180° or 240° windowed cylindrical light distributors in the oesophagus (provided by Medlight, Ecublens, Switzerland) (van den Bergh, 1986; van den Bergh et al, 1996).

The irradiance was checked before and after treatment and was set at 100 mW $cm^{-2}$ in order to avoid thermal damage. The exposure time was adapted so that the radiant exposure (total light dose) was 100 J $cm^{-2}$ at both wavelengths.

### Follow-up and interpretation of results

Patients were cautioned to avoid direct sunlight for 4–6 weeks after drug administration. A first endoscopic follow-up was performed 7–10 days after PDT to evaluate, macroscopically, the extent of the tumour necrosis. Subsequent endoscopies, with oesophageal vital staining (toluidine blue) as well as biopsies and abrasive or wash cytologies, were performed 3 months after the treatment and twice a year thereafter.

The patients were classified as having a complete response if the results at follow-up fulfilled all of the following criteria: (a) the endoscopic examination revealed no macroscopic tumour even after vital staining in the oesophagus; (b) all biopsy samples were negative for cancer; and (c) cytological washings or brushings showed no evidence of malignancy. Partial response was recorded when the endoscopy revealed no visible tumour but residual cancer persisted on biopsy or cytology. The treatment was considered a failure (no response) if tumour tissue was visualized at endoscopy and confirmed by biopsy.

### Histopathological studies

One surgical and two necropsy oesophageal specimens of patients previously treated by PDT for one, two and three distinct superficial cancers, respectively, were available for histopathological examination. The three specimens were available for analysis at 6, 12 and 36 months, respectively, after the last PDT session. None of these patients had received additional therapy by radiation or medication to these oesophageal tumours, and none of them had clinical or endoscopic evidence of gastro-oesophageal reflux disease. The whole specimen was fixed in 5% buffered formalin. Multiple serial sections (at least four) from each area treated by PDT as well as from the non-illuminated adjacent tissue were embedded in paraffin, processed in a routine manner and stained with haematoxylin and eosin, van Gieson and Masson's trichrome. The extent and severity of tissue damage and scarring were evaluated histologically by a senior pathologist who had no previous knowledge of the PDT parameters used. In addition, the thickness of the oesophageal wall (from the surface of the epithelium to the outer border of the muscularis propria) of each treated and adjacent non-irradiated areas was measured microscopically using a graded reticule. A total of ten measurements was recorded serially on each slide available for inspection.

**Figure 1** Long-term histological changes after PDT in the oesophagus. The samples are from sites 2 and 3 and from non-treated oesophagus in patient 1 as described in Table 2. (A) Non-treated area with normal histological architecture. E, epithelium; LP, lamina propria; LMM, lamina muscularis mucosae; SM, submucosa; MPI, muscularis propria interna; MPE, muscularis propria externa (H&E, 20 ×). (B) After two successive 514 nm PDTs to the site, scarring and fibrosis are restricted to the superficial layers (LP and SM) of the oesophageal wall as indicated by one asterisk. The LMM has been destroyed (Van Gieson, 20 ×). (C) After 630 nm PDT in an area previously treated with haematoporphyrin derivative and 630 nm light, there is disappearance of the normal architecture with marked thinning and transmural scarring (**) of the oesophageal wall (Van Gieson, 20 ×)

© Cancer Research Campaign 1998

### Statistical analysis

Means and standard errors of the means were calculated for the oesophageal wall thickness in the various sites examined. Comparison of the mean thickness between 514 nm and 630 nm irradiated areas as well as between treated and non-treated sites was performed using the Mann–Whitney *U*-test. A *P*-value < 0.01 was considered as significant.

### RESULTS

### Clinical results

Twenty-two superficial SCCs of the oesophagus (14 tumours) and the bronchi (eight tumours) were treated by PDT after Photofrin II sensitization. Considering all treatment variables, including the drug dose (1 or 2 mg $kg^{-1}$), drug light interval (1 h or 72 h) and wavelength (514 nm or 630 nm), the endoscopic controls 10 days after PDT revealed necrosis of the tumour and adjacent irradiated normal mucosa. There was no evidence of selectivity at the tissue surface, and the extent of the superficial tissue damage simply matched the geometry of the illuminated area.

The clinical results are presented in Table 1. Six of the nine (67%) superficial cancers treated with 514 nm light achieved a complete response after one (four tumours) or two (two tumours) PDT sessions. None of them had recurred after a mean follow-up of 21 months (range 6–49 months). A partial response was observed in the three remaining tumours.

In the 630 nm treatment group (13 tumours), complete response without relapse was recorded in nine cases (69%), with disease-free follow-up periods ranging from 6 to 85 months (mean 26 months). Cure was achieved after one PDT session in eight of the nine tumours and after two treatments 3 months apart in the last one. Three cancers achieved a partial response, and one exhibited only a minimal macroscopic reduction in size.

In the oesophagus, PDT with either green or red light was highly effective in eradicating in situ and intramucosal (T1a) cancer but failed to cure more than half of the submucosal (T1b) tumours.

Ten of the 26 PDTs were carried out on seven tumours (three tumours treated twice) at a drug–light interval of 1 h after Photofrin II injection. Although tumour tissue necrosis was observed at the first endoscopic control, only two of these tumours achieved a complete response (Table 1).

### Complications

No major complications, such as stenosis, perforation or fistula, were observed in either treatment group. However, three patients treated with red light reported severe chest pain, with associated high-grade fever for 10 days after PDT. Two of them, with early cancer in the lower oesophagus, had concomitant pleural effusion. The third, whose cancer was located on the left wall of the upper oesophagus, showed endoscopic evidence of oedema and erythema on the posterior wall of the trachea at the level of the oesophageal cancer. These three patients recovered fully under antimicrobial therapy. No such side-effects were noted after green light PDT.

### Histopathological study

Three specimens of oesophagus, each with either one, two or three distinct areas previously treated by PDT were available for study of the long-term tissue damage induced by PDT (Table 2). In each of the treated sites, healing and re-epithelialization were macroscopically complete. Microscopically, the wall of the non-PDT-treated oesophagus consisted of well distinguishable anatomical layers (Figure 1A); the lamina propria and submucosa consisted of loose connective tissue with scanty cellular components and blood vessels. In all of the treated areas, the epithelium was morphologically normal without residual cancer.

The three sites treated with 514 nm PDT showed evidence of scarring with collagen fibre deposits in the lamina propria and submucosa and disappearance of the lamina muscularis mucosae (Figure 1B). Focal areas of interstitial fibrosis and occasional atrophic smooth muscle fibres were also noted in the uppermost layers of the muscularis propria. However, the deeper layers of the muscularis propria did not display any morphological changes or fibrosis compared with non-treated sites. More dramatic changes were observed in the areas treated with red light. In two instances, the normal microscopic architecture of the oesophageal wall was

**Figure 2** Thickness of the oesophageal wall (mean ±s.e.) after PDT with 514 nm or 630 nm light for six distinct superficial cancers in the three patients reported in Table 2. Measurement of a non-irradiated area of the oesophagus of each patient is given as a control. *One previous PDT with haematoporphyrin derivative and 630 nm light to this site

 © Cancer Research Campaign 1998

completely blunted and replaced by dense collagen fibre deposition leading to transmural scarring (Figure 1C). In these two sites, only very few residual and markedly atrophic smooth muscle fibres were noted in the external part of the muscularis propria. Wherever present, the perioesophageal fat was also partially replaced by dense fibrosis. Although one of these two areas had received one previous PDT with HPD and red light, the second one displayed similar changes after a single treatment with Photofrin II and red light. Finally, in the third case, fibrous scarring completely replaced the muscularis propria interna. The externa was somewhat preserved but still exhibited some degree of interstitial fibrosis surrounding atrophic muscle fibres.

Compared with non-irradiated areas, PDT led to a significant ($P < 0.0001$) reduction of the oesophageal wall thickness in all but one of the green light-treated sites ($P = 0.09$) (Figure 2, patient 1). Furthermore, red light induced significantly ($P < 0.0001$) more pronounced thinning than did green light PDT, the reduction of the wall thickness being especially marked in the site previously treated with HPD-PDT and red light.

### DISCUSSION

Photodynamic therapy of cancer in the oesophagus and the tracheobronchial tree may occasionally result in severe complications, such as wall perforation, oesophago-tracheal fistula or stricture formation (Monnier et al, 1990; Hochain et al, 1993; Grosjean et al, 1996; McCaughan et al, 1996). Whereas massive tumour destruction may account for such a dramatic event after palliative PDT of a deep infiltrating cancer, transmural necrosis after PDT of a superficial carcinoma can only be explained by the non-selective damage of the healthy tissue underlying the tumour. This, in turn, is the result of the insufficient ratio between the PS concentration in the tumour and that in the underlying normal tissue, combined with the use of deep penetrating wavelengths of light.

In situ and microinvasive squamous cell carcinomas of the bronchi and the oesophagus are usually flat, slightly elevated or slightly depressed lesions, no thicker than 1 or 2 mm. For PDT of such tumours, the use of wavelengths that are less penetrating than the usual red light permits the selective illumination of only the superficial layers of the organ (Bays et al, 1996), thus avoiding the effect of insufficient selectivity of the PS distribution. Undesirable deep tissue damage can therefore be avoided, while keeping a high degree of PDT efficacy at lesser depths. Experimental animal studies have confirmed that HPD photosensitization with green light at 514 nm is very effective in inducing superficial tissue necrosis to depths of up to 2–3 mm (Bellnier et al, 1985; van Gemert et al, 1985). Our endoscopic examination 7–10 days after PDT showed superficial tissue necrosis in all instances without any real macroscopic difference in the amount of damage between the two treatment wavelengths. These observations are in agreement with those made by Nauta et al (1996) on the effects of green or red light on normal palatal mucosa in rats.

Clinically, our results show that PDT with Photofrin II and green light was highly effective in eradicating in situ and intramucosal (T1a) SCC in the oesophagus and that the complete response rate (100%) was equal to that obtained with red light. Results of PDT were, however, less satisfactory for bronchial and submucosal oesophageal cancer, with a slight trend towards better efficacy for red light treatment. Overall, the rate of complete response achieved with green light (67%) was not markedly different from the 69% of complete responses recorded after red light PDT. However, the small number of tumours in each treatment group does not allow the conclusion that the two wavelengths have completely equivalent efficacy. The results are, however, consistent with this hypothesis and are also in agreement with some preclinical and clinical reports from the literature showing that thin cancers can be controlled efficiently with either green or red light PDT (Bandieramonte et al, 1984; Foster et al, 1996).

No overt fistula was recorded in either treatment group. However, three patients treated with red light for oesophageal cancer exhibited severe side-effects (pain, fever and/or pleural effusion) that were interpreted as possible occult perforation. Interestingly, the histological analysis of the oesophagus of two of these patients (patients 1 and 3 in Table 2) displayed transmural scarring with marked thinning of the wall, thus confirming previous extensive tissue damage. It is also worth pointing out that in none of these three patients was the tumour located on the anterior wall of the upper oesophagus and thus just opposite to the posterior wall of the trachea. Outside of this critical location, transmural necrosis of the oesophagus may be confined by perioesophageal fat, thus leading to a covered perforation rather than to overt fistula.

Although only a few specimens were available for histological study, the results were consistent and showed that, whatever the treatment conditions and wavelengths used, PDT leads to some long-term localized tissue damage with fibrous scarring and thinning of the oesophageal wall. As the irradiance was always kept at 100 mW $cm^{-2}$, a concomitant thermal effect can certainly be ruled out (van Gemert and Welch, 1989), and the morphological changes observed must be considered the results of PDT only. The in-depth extent of the long-term tissue damage was, however, wavelength dependent: while full-thickness scarring and marked thinning of the oesophageal wall was evident after red light illumination, especially after two treatments at the same site, the fibrosis after one or two PDTs with green light never extended deeper than the most superficial layers of the muscularis propria. Thus, with green light, wall perforation was not expected to occur.

The contrasting effect of green and red light on the depth of scarring induced in the oesophageal wall is further emphasized if the respective Photofrin absorption coefficients (Bellnier et al, 1985) at both wavelengths used are considered. Taking this factor into account, it can be calculated that, for a given radiant energy fluence, the dose absorbed at 514 nm by a Photofrin II molecule is about three times greater than it is at 630 nm. Combining these factors with the optical properties of the oesophageal wall for 514 nm and 630 nm respectively (Bays et al, 1997), it can be estimated that, in our setting, the 100 J $cm^{-2}$ green light radiant exposure (total light dose) was equivalent to approximately 235 J $cm^{-2}$ of red light. Nevertheless, and owing to the greater absorption by the tissue of green light relative to red light (Bays et al, 1997), the damage in the areas treated with 514 nm PDT was always limited to the upper layers of the oesophageal wall. This simply underlines that the use of green light greatly enhances the 'in-depth' necrosis selectivity as well as the safety of PDT for the treatment of superficial cancer in the oesophagus.

Finally, and although this was not the main purpose of the present study, ten PDTs were carried out as early as 1 h, instead of 72 h, after the injection of Photofrin II. This was motivated by a previous in vivo fluorescence pharmacokinetic study in patients with early SCC in the upper aerodigestive tract, which showed that, although the Photofrin II fluorescence in both tumour and normal mucosa reached a maximum intensity between 10 and 15 h after injection and plateaued thereafter, the highest fluorescence

© Cancer Research Campaign 1998

ratio between early cancer and adjacent normal mucosa was observed within the first hour after the injection (Braichotte et al, 1995). It was therefore hoped that this improved fluorescence contrast would result in better therapeutic selectivity. Selective tumour necrosis was, however, never observed, no matter what drug–light interval was used. Performing PDT 1 h after Photofrin II injection was, however, associated with a poor outcome, and only two out of the seven superficial cancers treated achieved a complete response. Two possible explanations can be put forward. First, at such a short drug–light interval, most of the injected Photofrin is probably located in or near the vasculature, and vascular shutdown only, without associated direct phototoxic cellular kill (Henderson and Dougherty, 1992), is probably to be expected after the administration of the light. As early SCC are only poorly or not at all vascularized, this vascular damage alone may be insufficient to eradicate the tumour. Secondly, the photodynamic dose at the tumour bed may have been insufficient at this short drug–light interval. This is substantiated by the fact that, at very short delays after injection, the intensity of the fluorescence signal of Photofrin II, which is known to correlate more or less with the drug concentration, is much lower than at 72 h after the drug administration (Braichotte et al, 1995). The low concentration of the dye in the tumour combined with attenuation of the incident light may be insufficient to reach the photodynamic threshold in the deeper part of the tumour, thus leading to incomplete response.

In summary, our study suggests that, for the curative treatment of in situ (Tis) and intramucosal (T1a, N0 stage) cancer in the oesophagus, green light PDT with Photofrin II permits specific damaging of only the superficial layers of the wall and thus greatly reduces the risk of perforation, while maintaining a high degree of efficacy. For identical indications, very good clinical results have also been obtained with green light after photosensitization with the second-generation photosensitizer m-tetra(hydroxyphenyl) chlorin (Grosjean et al, 1996; Savary et al, 1997). In the tracheobronchial tree, except for the membranous part of the trachea and the main bronchi, the presence of cartilage, which takes up little PS (Smith et al, 1993; Andrejevic et al, 1996), renders perforation almost impossible. In these cases, green light may offer no advantage over red light.

### ACKNOWLEDGEMENTS

This work was supported by the Swiss National Fund (PN 18) the CHUV-EPFL-UNIL fund for collaboration in the area of biomedical technology, the 'Fonds de Service' and 'Fonds de Perfectionnement' of the Otolaryngology, Head and Neck Surgery Department, CHUV Hospital, the Swiss National Priority Program in Optics, as well as by the Swiss Commission for Technology and Innovation. We are also grateful to Quadra Logic Technology Inc. (Vancouver, BC, Canada) for kindly providing the Photofrin II.

### REFERENCES

Akaogi E, Ogawa I, Mitsui K, Onizuka M, Ishikawa S, Yamamoto T, Inage Y and Ogata T (1994) Endoscopic criteria of early squamous cell carcinoma of the bronchus. *Cancer* **74**: 3113–3117

Andrejevic S, Savary J-F, Monnier P, Brachotte D, Wagnieres G and van den Berg H (1996) Measurement by fluorescence microscopy of the time dependent meso-tetrahydroxyphenyl chlorin distribution in healthy tissues and chemically-induced 'early' squamous cell carcinoma of the Syrian hamster cheek pouch. *J Photochem Photobiol B Biol* **36**: 143–151

Bandieramonte G, Marchesini R, Melloni E, Andreoli C, Di Pietro S, Spinelli P, Fava G, Zunino F and Emanuelli H (1984) Laser phototherapy following HpD administration in superficial neoplastic lesions. *Tumori* **70**: 327–334

Barr H, Shepherd NA, Dix A, Roberts DJ, Tan WC and Krasner N (1996) Eradication of high-grade dysplasia in columnar-lined (Barrett's) oesophagus by photodynamic therapy with endogenously generated protoporphyrin IX (see comments). *Lancet* **348**: 584–585

Bays R, Wagnieres G, Robert D, Braichotte D, Savary JF, Monnier P and van den Bergh H (1996) Clinical determination of tissue optical properties by endoscopic spatially resolved reflectometry. *Appl Optics* **35**: 1756–1766

Bays R, Wagnieres G, Robert D, Braichotte D, Savary JF, Monnier P and van den Bergh H (1997) Light dosimetry for photodynamic therapy in the esophagus. *Lasers Surg Med* **20**: 290–303

Bellnier DA, Prout GR, Jr and Lin CW (1985) Effect of 514.5-nm argon ion laser radiation on hematoporphyrin derivative-treated bladder tumor cells in vitro and in vivo. *J Natl Cancer Inst* **74**: 617–625

Bown SG (1993) Photodynamic therapy in gastroenterology – current status and future prospects (review). *Endoscopy* **25**: 683–685

Braichotte DR, Wagnieres GA, Bays R, Monnier P and van den Bergh HE (1995) Clinical pharmacokinetic studies of photofrin by fluorescence spectroscopy in the oral cavity, the esophagus, and the bronchi. *Cancer* **75**: 2768–2778

Cortese DA, Edell ES and Kinsey JH (1997) Photodynamic therapy for early stage squamous cell carcinoma of the lung. *Mayo Clin Proc* **72**: 595–602

Delaney TF, Sindelar WF, Tochner Z, Smith PD, Friauf WS, Thomas G, Dachowski L, Cole JW, Steinberg SM and Glatstein E (1993) Phase I study of debulking surgery and photodynamic therapy for disseminated intraperitoneal tumours. *Int J Radiat Oncol Biol Phys* **25**: 445–457

Fisher AM, Murphree AL and Gomer CJ (1995) Clinical and preclinical photodynamic therapy (review). *Lasers Surg Med* **17**: 2–31

Foster TH, Gibson SL and Raubertas RF (1996) Response of Photofrin-sensitised mesothelioma xenografts to photodynamic therapy with 514 nm light. *Br J Cancer* **73**: 933–936

Grosjean P, Savary J, Wagnières G, Mizeret J, Woodtli A, Theumann J, Fontolliet C, van den Berg H and Monnier P (1996) Tetra(m-hydroxyphenyl)chlorin clinical photodynamic therapy of early bronchial and esophageal cancer. *Lasers Med Sci* **11**: 227–235

Hayata Y, Kato H, Furuse K, Kusunoki Y, Suzuki S and Mimura S (1996) Photodynamic therapy of 168 early stage cancers of the lung and oesophagus: a Japanese multi-centre study. *Lasers Med Sci* **11**: 255–259

Henderson B and Dougherty T (1992) How does photodynamic therapy work? *Photochem Photobiol* **61**: 397–401

Hochain P, Ducrotte P, Touchais J, Paillot B and Hecketsweiler P (1993) Extended necrosis of the oesophageal wall after photodynamic therapy: report of two cases. *Lasers Med Sci* **8**: 147–150

McCaughan JS, Jr, Ellison EC, Guy JT, Hicks WJ, Jones JJ, Laufman LR, May E, Nims TA, Spiridonidis CH and Williams TE (1996). Photodynamic therapy for esophageal malignancy: a prospective twelve-year study. *Ann Thorac Surg* **62**: 1005–1009; discussion 1009–1010.

Monnier P, Savary M, Fontolliet C, Wagnières G, Châtelain A, Cornaz P, Depeursinge C and van den Berg H (1990) Photodetection and photodynamic therapy of 'early' squamous cell carcinomas of the pharynx, oesophagus and tracheo-bronchial tree. *Lasers Med Sci* **5**: 149–169

Monnier P, Fontolliet C and Ollyo J (1994) Endoscopic findings in 100 early-stage esophageal cancers. *Diagn Therap Endosc* **1**: 83–92

Nauta JM, Van Leengoed HLLM, Witjes MJH, Roodenburg JLN, Nikkels PGJ, Thomsen SL, Marijnissen JPA and Star WM (1996) Photofrin-mediated photodynamic therapy of rat palatal mucosa: normal tissue effects and light dosimetry. *Lasers Med Sci* **11**: 163–174

Nseyo UO, Whalen RK and Lundahl SL (1993) Canine bladder response to red and green light whole bladder photodynamic therapy. *Urology* **41**: 392–396

Overholt BF and Panjehpour M (1996) Photodynamic therapy for Barrett's esophagus: clinical update. *Am J Gastroenterol* **91**: 1719–1723

Savary J-F, Monnier P, Fontolliet C, Mizeret J, Wagnières G, Braichotte D and van den Bergh H (1997) Photodynamic therapy for early squamous cell carcinomas of the esophagus, bronchi, and mouth with m-tetra (hydroxyphenyl) chlorin. *Arch Otolaryngol Head Neck Surg* **123**: 162–168

Sibille A, Lambert R, Souquet JC, Sabben G and Descos F (1995) Long-term survival after photodynamic therapy for esophageal cancer. *Gastroenterology* **108**: 337–344

Smith SG, Bedwell J, Macrobert AJ, Griffiths MH, Bown SG and Hetzel MR (1993) Experimental studies to assess the potential of photodynamic therapy for the treatment of bronchial carcinomas (see comments). *Thorax* **48**: 474–480

Stables GI and Ash DV (1995) Photodynamic therapy (Review). *Cancer Treat Rev* **21**: 311–323

© Cancer Research Campaign 1998

van den Bergh H (1986) Light and porphyrins in cancer therapy. *Chem Brit* **22**: 430–439

van den Bergh H, Mizeret J, Theumann J-F, Woodtli A, Bays R, Robert D, Thielen P, Philippoz J-M, Braichotte D, Forrer M, J-F, S, Monnier P and Wagnieres G (1996) Light distributors for photodynamic therapy. *SPIE Proc Series* **2631**: 173–198

van Gemert MJ and Welch AJ (1989) Time constants in thermal laser medicine. *Lasers Surg Med* **9**: 405–421

van Gemert JC, Berenbaum MC and Gijsbers GH (1985) Wavelength and light-dose dependence in tumour phototherapy with haematoporphyrin derivative. *Br J Cancer* **52**: 43–49

Wilson BC, Jeeves WP and Lowe DM (1985) In vivo and post mortem measurements of the attenuation spectra of light in mammalian tissues. *Photochem Photobiol* **42**: 153–162

© Cancer Research Campaign 1998